# Immediate effect of gait training using the hybrid assistive limb in patients in the acute phase of stroke: a prospective pilot study

**DOI:** 10.3389/fnbot.2026.1985500

**Published:** 2026-10-23

**Authors:** Taku Matsuda, Shin Kondo, Hiroshi Kagusa, Masaaki Korai, Takeshi Miyamoto, Ryoma Morigaki, Kenji Shimada, Nori Sato, Kimiko Umemura, Tetsuya Matsuura, Yuishin Izumi, Shinsuke Katoh, Yasushi Takagi

**Affiliations:** 1 Department of Neurosurgery, Graduate School of Biomedical Sciences, Tokushima University, Tokushima, Japan; 2 Division of Rehabilitation, Tokushima University Hospital, Tokushima, Japan; 3 Department of Rehabilitation Medicine, Tokushima University Hospital, Tokushima, Japan; 4 Department of Neurology, Graduate School of Biomedical Sciences, Tokushima University, Tokushima, Japan

**Keywords:** acute phase of stroke, gait training, hybrid assistive limb, immediate effect, rehabilitation, robot

## Abstract

**Introduction:**

The effectiveness of Hybrid Assistive Limb (HAL) gait training in patients in the acute phase of stroke remains unclear. We prospectively assessed the immediate effects of HAL gait training in this patient population.

**Methods:**

In this prospective pilot study, four patients with acute-phase stroke undergoing HAL gait training were included. The 10-meter walking test (10MWT) at maximum speed was conducted immediately before and after HAL or conventional gait training sessions. Step length, velocity, and cadence during the 10MWT and their changes from before to after training were evaluated. Differences in these changes between HAL and conventional gait training were analyzed after adjustment for patient-specific differences, the corresponding pre-training value, and days from the first gait-training session. Correlations between pre-training values and rates of change were also analyzed. In an exploratory subgroup analysis, sessions were categorized according to pre-training velocity as slower (S; ≤0.4 m/s) or faster (F; >0.4 m/s). Rates of changes in gait parameters were compared between HAL and conventional training across all units and within each group.

**Results:**

After adjustment, no significant differences in gait parameter changes were observed between HAL and conventional gait training. The adjusted between-condition differences (HAL − conventional) were 0.033 m/s for velocity (95% confidence interval [CI], −0.004 to 0.071; *p* = 0.0775), 1.60 cm for step length (95% CI, −0.05 to 3.25; *p* = 0.0564), and 2.02 steps/min for cadence (95% CI, −2.10 to 6.14; *p* = 0.3278). Immediately after HAL training, step length (*p* < 0.0001) and velocity (*p* = 0.0169) increased significantly, whereas cadence remained unchanged (*p* = 0.5848). No significant changes were observed after conventional training. Pre-training cadence negatively correlated with its rate of change after HAL training. In the exploratory S-group analysis, rates of change in step length, velocity, and cadence were higher after HAL than after conventional training (*p* = 0.0103, 0.0057, and 0.0261, respectively).

**Discussion:**

Although the adjusted analysis showed no significant differences between HAL and conventional gait training, HAL training was associated with immediate improvements in step length and walking velocity in the within-condition analysis. Exploratory findings suggest that patients with slower walking velocity may show a greater immediate response to HAL gait training. Further studies are warranted to confirm these findings.

## 1 Introduction

Recovery of ambulatory ability is a key therapeutic objective in patients with stroke without significant consciousness or cognitive disturbances. Robot-assisted rehabilitation has been introduced to achieve better recovery than that provided by conventional rehabilitation (Warutkar et al., 2022), and various types of robots have been developed (Zhang et al., 2017; Selves et al., 2020).

The Hybrid Assistive Limb (HAL; Cyberdyne Inc., Ibaraki, Japan) is a wearable exoskeleton device developed to improve the physical function of patients (Morishita and Inoue, 2016; Saita et al., 2018). There are three types of HALs: lower-limb, single-joint, and lumbar (Fujikawa et al., 2022). The HAL supports movement by detecting weak bioelectrical signals (BESs) from the skin surface that are generated by the wearer’s intention to move. Information about movement is sent to the brain as sensory signals, thereby enhancing output from the central nervous system. This loop, referred to as “interactive biofeedback,” may be activated during HAL gait training, promoting neuroplasticity and recovery from hemiparesis, which are potential benefits of HAL gait training (Morishita and Inoue, 2016; Saita et al., 2018).

The HAL has been progressively adopted in the rehabilitation of patients with diverse clinical conditions, such as neurological disorders, spinal cord injuries, and stroke. Consistent with current clinical guidelines, robot-assisted movement therapy—when combined with conventional rehabilitation approaches—is recommended to enhance motor performance and functional mobility in individuals recovering from stroke (Winstein et al., 2016). Recent clinical studies have suggested that HAL may facilitate motor recovery in patients with stroke, particularly during the subacute and chronic phases of rehabilitation (Nilsson et al., 2014; Wall et al., 2015; Mizukami et al., 2016; Tanaka et al., 2019; Taki et al., 2020; Palmcrantz et al., 2021).

The therapeutic potential of HAL gait training in patients in the acute phase of stroke remains to be fully elucidated (Fukuda et al., 2015). Some studies have found no significant differences in functional outcomes when comparing HAL-assisted gait training with conventional training in these patients (Ogata et al., 2015; Yokota et al., 2023). However, Ogata et al. (2015) reported better outcomes in the HAL gait training group than in the conventional group of patients with right intracranial hemorrhage. In a previous study, the Apathy Scale scores decreased in the HAL gait training group, suggesting that HAL could promote early mobilization in patients with stroke (Yokota et al., 2023). Additionally, early initiation of HAL gait training has been associated with better outcomes (Degami et al., 2022). Notably, these studies evaluated the long-term outcomes of early initiation of HAL gait training.

At our institution, we conduct gait training with HAL in patients in the acute phase of stroke and often observe improvements in walking ability immediately following training, which we refer to as the “immediate effect” of HAL gait training. Although some case reports have suggested this effect in patients with cerebral palsy (Matsuda et al., 2018; Nakagawa et al., 2020), to the best of our knowledge, no such findings have been reported in patients with stroke. Therefore, in this study, we aimed to clarify the immediate effect of HAL gait training in patients in the acute phase of stroke using objective parameters, compared with conventional gait training.

## 2 Materials and methods

### 2.1 Study design and participants

In this prospective pilot study, patients who met our criteria and underwent HAL gait training at Tokushima University Hospital between January 2021and August 2021 for hemiparesis due to cerebral hemorrhage or infarction were enrolled.

Written informed consent was obtained from all patients. This study was conducted in accordance with the tenets of the Declaration of Helsinki and approved by the Ethics Committee of Tokushima University Hospital (approval number: 3123).

### 2.2 Inclusion criteria

The inclusion criteria for the HAL gait training program were as follows: (1) age < 80 years; (2) stroke onset within the past 7 days; (3) a Functional Ambulation Category (FAC) of 0–2 at the start of rehabilitation and an FAC of 4–5 before stroke onset; and (4) BESs detectable by the HAL.

### 2.3 Exclusion criteria

The exclusion criteria were as follows: (1) consciousness disorders, sensory aphasia, or cognitive dysfunction; (2) spasticity in the lower limbs; (3) a Modified Ashworth Scale score of 1 or higher; (4) active orthopedic, respiratory, or cardiovascular disease; or (5) dermatological conditions that prevent electrode attachment to the skin.

### 2.4 Protocol

The rehabilitation timeline is shown in Figure 1. Conventional gait training was started the day after stroke onset, and the HAL gait training program was initiated 1 week after stroke onset. During physiotherapy, patients underwent gait training for 20 min (one unit), twice daily, 5 days per week, for 3 weeks. During the 3 weeks, HAL gait training was conducted for three units per week (total: nine units), with conventional gait training provided for the remaining units (Figure 1). A physiotherapist and a neurosurgeon assisted the patients during HAL gait training. The Cybernic Voluntary Control mode, which assists patients’ movements based on BESs generated by their voluntary movements, was used on both sides, and other HAL settings were adjusted by the therapist according to each patient’s gait condition. A mobile personal lifter, All-In-One Walking Trainer (Ropox A/S, Ringstedgade, Denmark), was used when necessary. Safety was monitored throughout the training sessions by recording the occurrence of falls, electrode-related skin injury, abnormal vital signs, and training interruptions.

**Figure 1:**
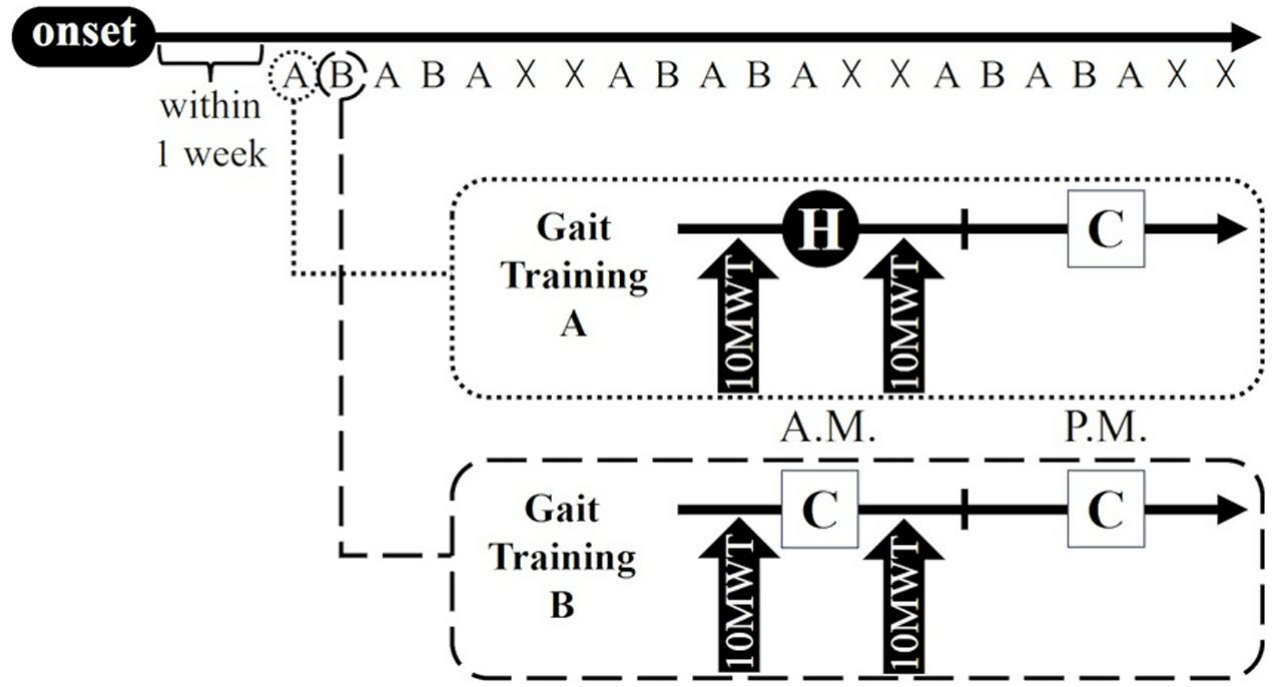
Protocol for rehabilitation. Rehabilitation was initiated within 1 week of stroke onset. In gait training A, HAL gait training was conducted in the morning (AM) and conventional gait training was conducted in the afternoon (PM). In gait training B, only conventional gait training was conducted both in the morning and afternoon. These two gait trainings were performed alternately on weekdays for 3 weeks. C, conventional gait training; H, HAL gait training; HAL, Hybrid Assistive Limb; 10MWT, 10-meter walking test.

When patients were able to walk with a cane or assistance, they underwent a 10-meter walking test (10MWT) at maximum speed immediately before and after each gait training. The post-training 10MWT was performed within 5 min after the completion of each gait training session. For 10MWT, 2-m acceleration and deceleration zones were provided before and after the 10-m measurement section. The test was performed twice, and the faster trial was used for analysis. Measurements were limited to the morning gait training sessions to minimize the potential effects of fatigue and cumulative exertion from earlier training on the afternoon sessions. Time and the number of steps were recorded, and step length, velocity, and cadence were calculated. Step length was calculated as the total walking distance divided by the number of steps. Cadence was calculated as the number of steps divided by walking time and expressed as steps per minute. Orthotic support and the level of assistance were reduced as appropriate in accordance with improvements in motor function over the course of recovery, while ensuring consistency between the pre- and post-training measurements within each training unit. Measurements were conducted by an assessor blinded to the training conditions. The units in which the 10MWT was conducted are shown in Figure 1.

Changes in step length, velocity, and cadence during each 10MWT between immediately before and after HAL or conventional gait training were evaluated. The absolute changes in these gait parameters (post-training minus pre-training) were compared between HAL and conventional gait training using the adjusted linear models described below. Correlations between gait parameters (step length, velocity, and cadence) immediately before gait training and their respective rates of change immediately after gait training were examined to clarify the patient characteristics associated with the observed immediate effect.

According to a previous report, patients with a walking velocity of less than 0.4 m/s were considered limited to household-level walking (Perry et al., 1995). Based on this threshold, the units were categorized into two groups: slower (S) (≤ 0.4 m/s) and faster (F) (> 0.4 m/s). As exploratory analyses, rates of change were compared between HAL and conventional gait training across all units, as well as within the F and S groups.

### 2.5 Statistical analysis

Statistical analyses were conducted using JMP software (version 14.0.0; SAS Institute Inc., Cary, NC, USA). To compare changes in gait parameters between HAL-assisted and conventional gait training, linear models were constructed for changes in gait velocity, step length, and cadence, accounting for patient-specific differences and potential confounding factors. Training condition (HAL-assisted or conventional), patient ID, the corresponding pre-training value, and days from the first gait-training session were included as fixed effects. Adjusted between-condition differences (HAL minus conventional) were estimated using least-squares means and are presented with 95% confidence intervals (CIs). For the other analyses, the distribution of each dataset was evaluated using the Shapiro–Wilk test to assess normality. As the data did not follow a normal distribution, nonparametric methods were employed. The Wilcoxon signed-rank test was applied for paired comparisons, whereas the Mann–Whitney U test was used to assess differences between independent groups. Associations between variables were examined using Spearman’s rank correlation coefficient. Statistical significance was set at *p* < 0.05.

## 3 Results

The clinical profiles of the four patients enrolled in this study are detailed in Table 1. No adverse events, including falls, electrode-related skin injuries, abnormal vital signs, or training interruptions, occurred during the gait training sessions. Figure 2 shows the time course of changes in gait parameters for each patient. Individual session-level data for all patients are shown in Supplementary Table 1. After adjustment for patient-specific differences, the corresponding pre-training value, and days from the first gait-training session, no significant differences were observed in changes in gait parameters between HAL and conventional gait training. The adjusted between-condition difference (HAL − conventional) in walking velocity was 0.033 m/s (95% CI, −0.004 to 0.071; *p* = 0.0775). The corresponding difference in step length was 1.60 cm (95% CI, −0.05 to 3.25; *p* = 0.0564), and that in cadence was 2.02 steps/min (95% CI, −2.10 to 6.14; *p* = 0.3278) (Table 2).

**Table 1:** Patient characteristics.

Case	Age (years)	Sex	Diagnosis	Location	Pre-rehabilitation				One month after onset		
					BRS-LE	NIHSS	mRS	BI	NIHSS	mRS	BI
Case 1	52	F	Hemorrhage	Rt. putamen	III	9	4	20	3	4	70
Case 2	69	M	Infarction	Lt. corona radiata	I	13	4	20	8	4	60
Case 3	76	M	Infarction	Rt. parietal	V	4	4	40	1	2	100
Case 4	74	M	Infarction	Lt corona radiata	III	6	5	15	2	4	65

Rt., right; Lt., Left; BRS-LE, Brunnstrom recovery stage for the lower extremities; NIHSS, National Institutes of Health Stroke Scale; mRS, modified Rankin Scale; BI, Barthel Index; M, male; F, female.

**Figure 2:**
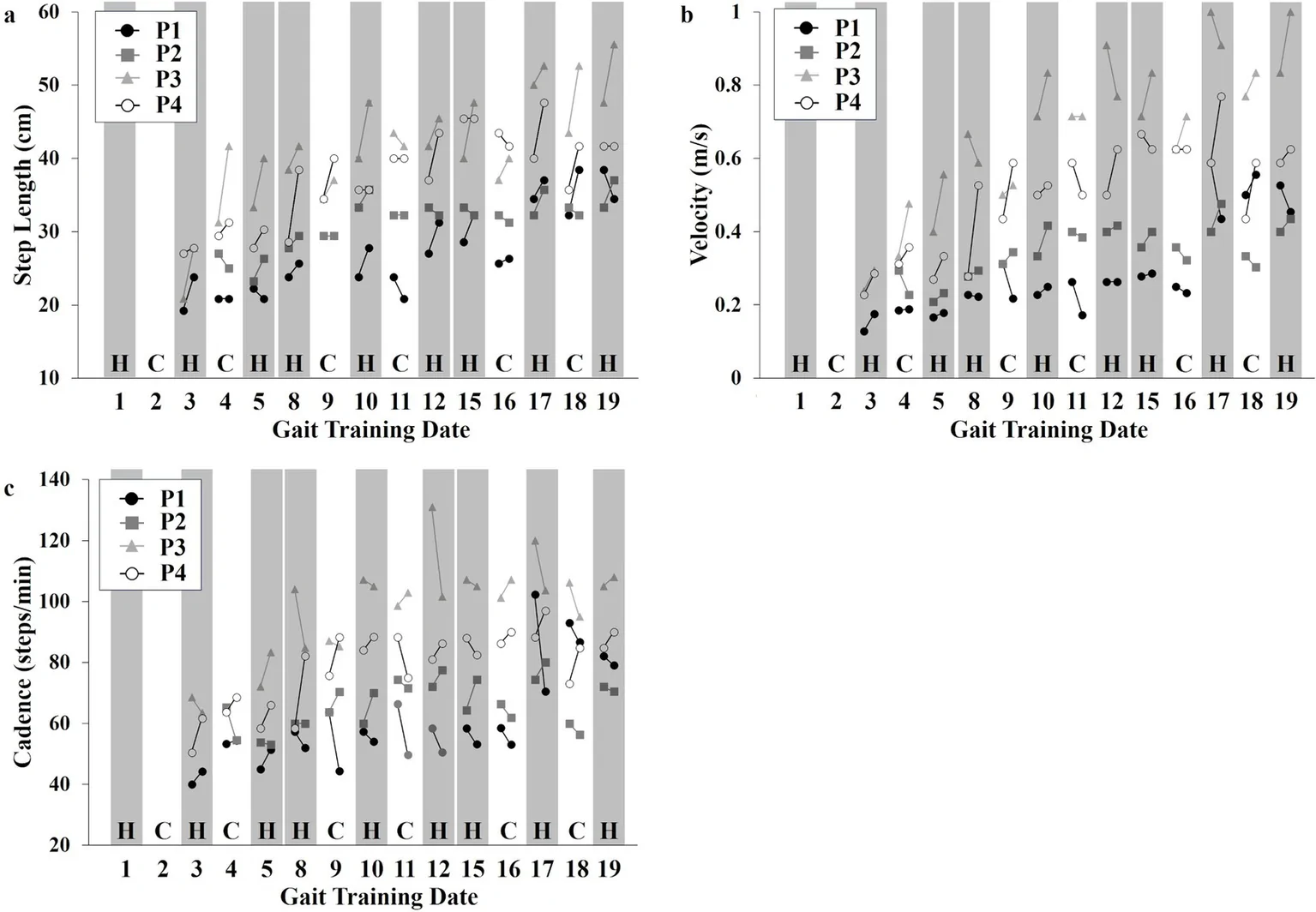
Time course of changes in gait parameters for each patient. **(a)** Step length, **(b)** velocity, and **(c)** cadence. C, conventional gait training; H, HAL gait training; P, Patient.

**Table 2:** Adjusted differences in changes in gait parameters between HAL and conventional gait training.

Outcome	Adjusted difference (HAL − Conventional)	95% CI	*p* value
Δ gait velocity, m/s	0.033	−0.004–0.071	0.078
Δ step length, cm	1.6	−0.05–3.25	0.056
Δ cadence, steps/min	2.02	−2.10–6.14	0.328

Between-condition differences were calculated as HAL minus conventional gait training. Analyses were adjusted for days from the first gait-training session and accounted for repeated measurements within patients. CI, confidence interval; HAL, Hybrid Assistive Limb.

The 10MWT was conducted in 60 units (Figure 3), and nine gait-training units (five HAL and four conventional) were excluded because the patients were unable to walk with a cane or assistance. The remaining 51 units (31 HAL and 20 conventional gait training units) were evaluated (Figure 3). No significant differences were observed in walking ability immediately before gait training between the HAL and conventional gait training groups (Supplementary Table 2).

**Figure 3:**
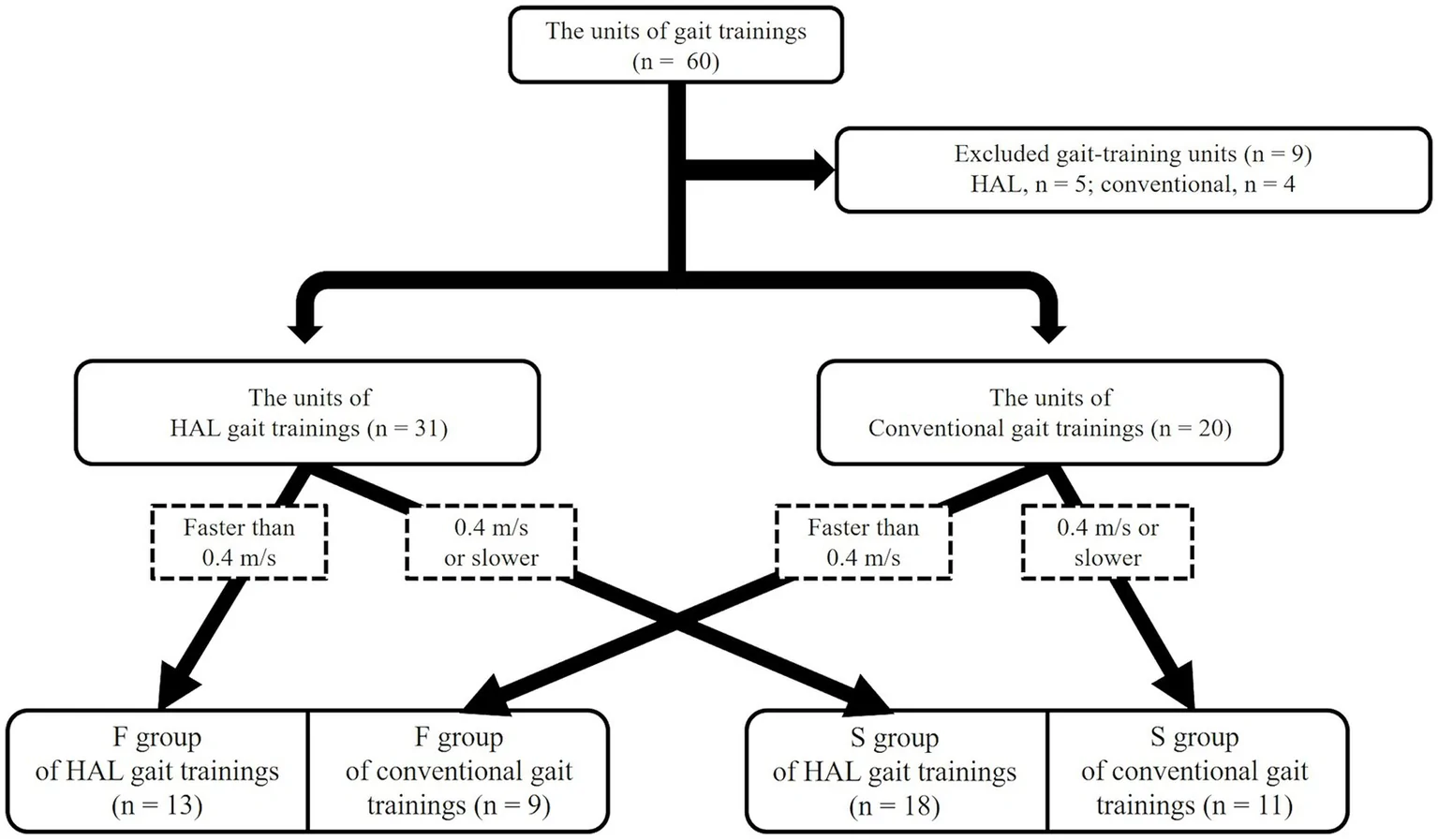
Flow chart of the units of gait training. Of 60 units assessed with the 10-Meter Walk Test, nine units (five HAL and four conventional gait training units) were excluded because the patients were unable to walk with a cane or assistance. The final analysis included 31 HAL units and 20 conventional gait training units. Among these, 13 HAL units and 9 conventional units were in the > 0.4 m/s gait speed group, while 18 HAL units and 11 conventional units were in the ≤ 0.4 m/s gait speed group. F, faster; S, slower; HAL, Hybrid Assistive Limb.

Changes in step length, velocity, and cadence immediately before and after gait training are shown in Figure 4. Immediately after HAL gait training, step length and velocity improved significantly (step length, *p* < 0.0001; velocity, *p* = 0.0169), whereas cadence remained unchanged (*p* = 0.5848) (Figure 4a). However, none of these parameters changed significantly immediately after conventional gait training (step length, *p* = 0.1670; velocity, *p* = 0.4820; and cadence, *p* = 0.4980) (Figure 4b).

**Figure 4:**
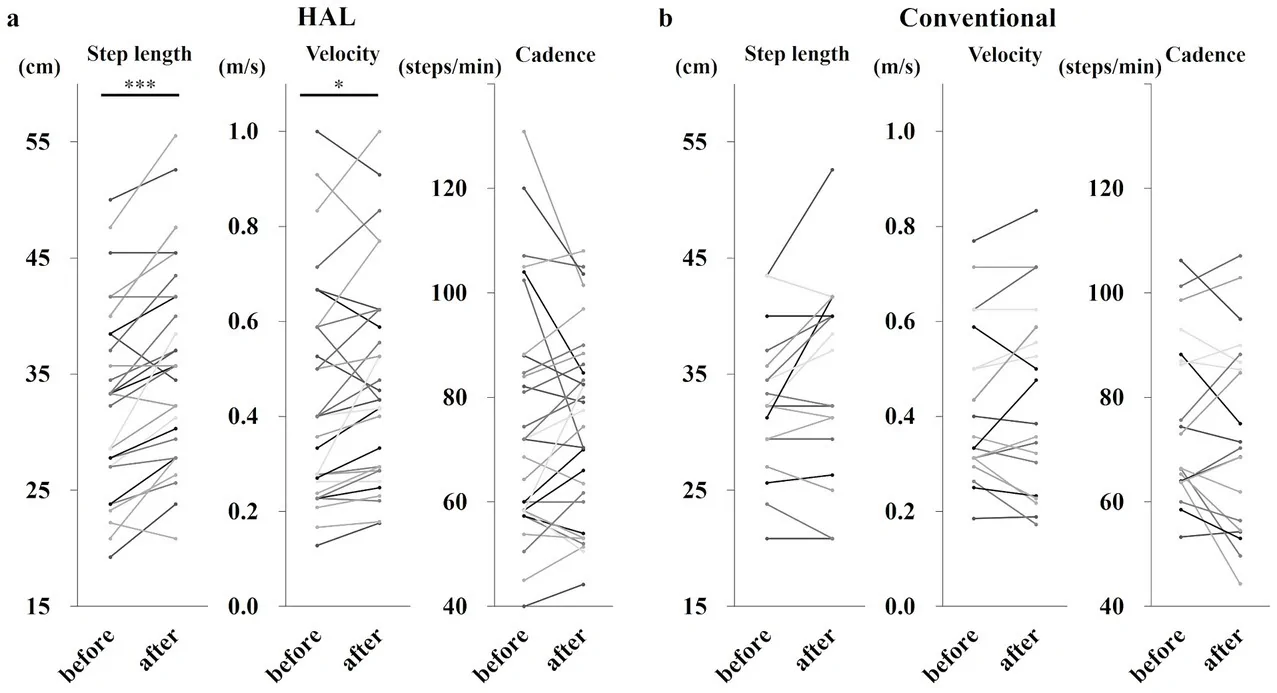
Changes in step length, velocity, and cadence between immediately before and after gait training. HAL gait training significantly improved step length and gait velocity but not cadence, whereas conventional gait training resulted in no significant changes in any parameter. **(a)** HAL gait training, **(b)** conventional gait training. *p < 0.05; ***p < 0.001; Wilcoxon’s signed-rank test. HAL, Hybrid Assistive Limb.

Figure 5 shows the correlations between the measurements obtained immediately before gait training and the rates of change immediately after gait training. In the HAL gait training group, a significant negative correlation was found between cadence and its rate of change (ρ = −0.4176, *p* = 0.0194), whereas no significant correlations were observed for step length or velocity (step length, ρ = −0.1730, *p* = 0.3520; velocity, ρ = −0.3268, *p* = 0.0728) (Figure 5a). In contrast, in the conventional gait training group, no significant correlations were found between any of the gait parameters and their rates of change (step length, ρ = 0.2026, *p* = 0.3917; velocity, ρ = 0.3058, *p* = 0.1898; and cadence, ρ = 0.0264, *p* = 0.9121) (Figure 5b).

**Figure 5:**
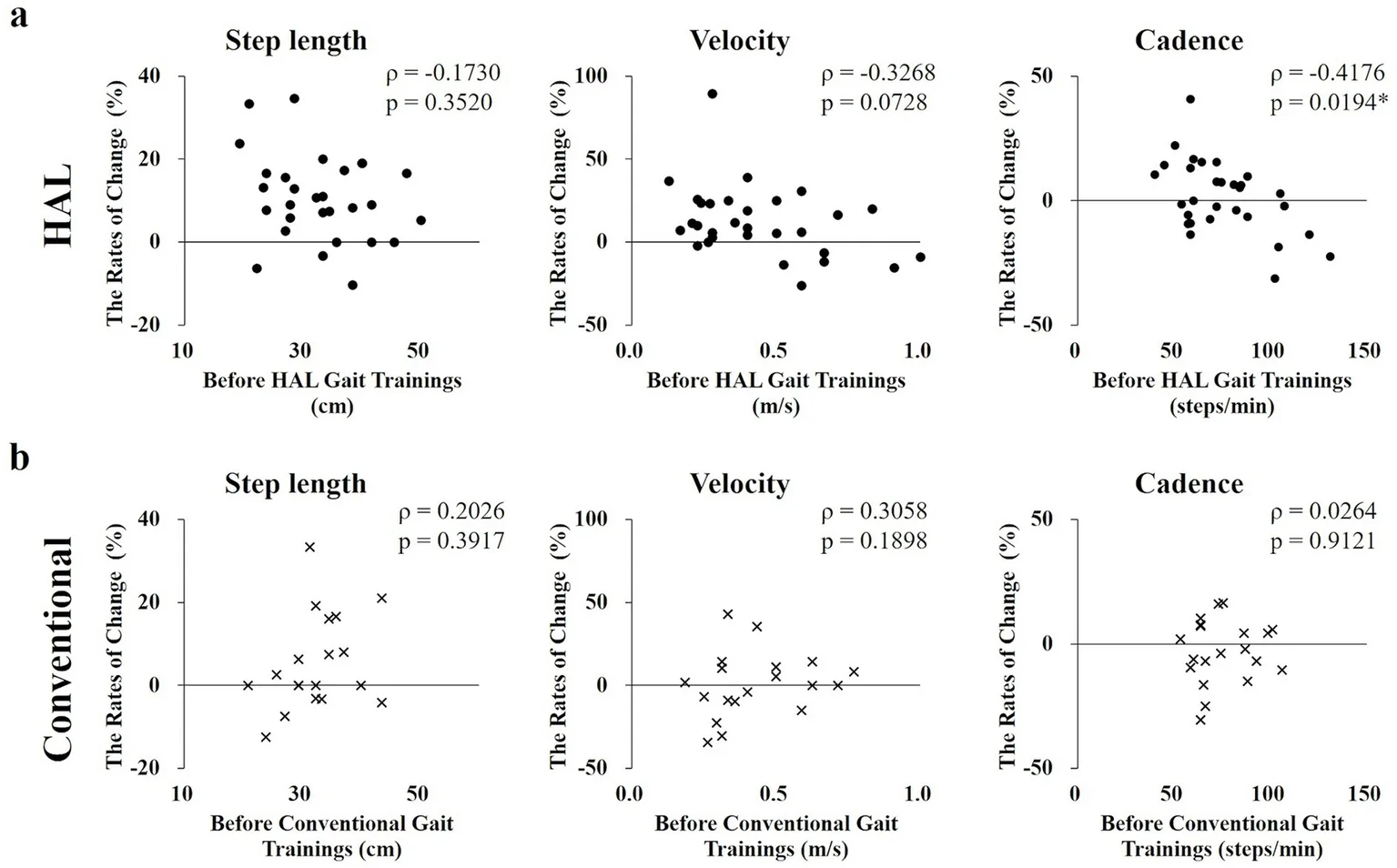
Correlations between step length, velocity, and cadence immediately before and change rates after gait training. A significant negative correlation was observed only for cadence in the HAL gait training group, whereas no significant correlations were found for step length or gait velocity. No significant correlations were observed for any parameter in the conventional gait training group. **(a)** HAL gait training, **(b)** conventional gait training. The correlation coefficient (ρ) and corresponding *p*-values (p) are shown. *p < 0.05, Spearman’s rank correlation coefficient. HAL, Hybrid Assistive Limb.

The comparisons between HAL and conventional gait training for each rate of change immediately after gait training are shown in Table 3 and Figure 6. Changes in velocity over time and the classification of units into the S and F groups for each patient are shown in Figure 2b and Supplementary Table 3, respectively. Among all units, no significant differences were found in any of the evaluated parameters between HAL and conventional gait training (step length, *p* = 0.0508; velocity, *p* = 0.0990; and cadence, *p* = 0.2886) (Figure 6a). Significant differences were observed in all measured parameters within the S group between the gait training methods (step length, *p* = 0.0103; velocity, *p* = 0.0057; and cadence, *p* = 0.0261) (Figure 6b). In contrast, in the F group, no significant differences were observed in any of the parameters between the gait training methods (step length, *p* = 1.0000; velocity, *p* = 0.4420; and cadence, *p* = 0.3847) (Figure 6c).

**Table 3:** Rates of change immediately after conventional and HAL gait training in the total, S, and F groups.

Gait parameters	Total			S group			F group		
	HAL (*n* = 31)	Conventional (*n* = 20)	*p*-value	HAL (*n* = 18)	Conventional (*n* = 11)	*p*-value	HAL (*n* = 13)	Conventional (*n* = 9)	*p*-value
Step length (%)	9.1 (−10.3–34.6)	0.0 (−12.5–33.3)	0.0508	10.9 (−6.3–34.6)	0.0 (−12.5–33.3)	0.0103*	8.3 (−10.3–19.0)	8.0 (−4.2–21.1)	1.0000	Velocity (%)	10.0 (−26.1–89.5)	1.0 (−34.5–42.9)	0.0990	11.8 (−2.2–89.5)	−7.0 (−34.5–42.9)	0.0057**	5.3 (−26.1–30.8)	8.3 (−15.0–35.3)	0.4420	Cadence (%)	0.0 (−31.2–40.8)	−2.9 (−30.4–16.6)	0.2886	7.6 (−13.5–40.8)	−6.1 (−30.4–10.3)	0.0261*	−2.0 (−31.2–9.8)	4.3 (−15.0–16.6)	0.3847

*p < 0.05; **p < 0.01; Mann–Whitney’s U-test.

S, slow; F, fast; HAL, Hybrid Assistive Limb.

**Figure 6:**
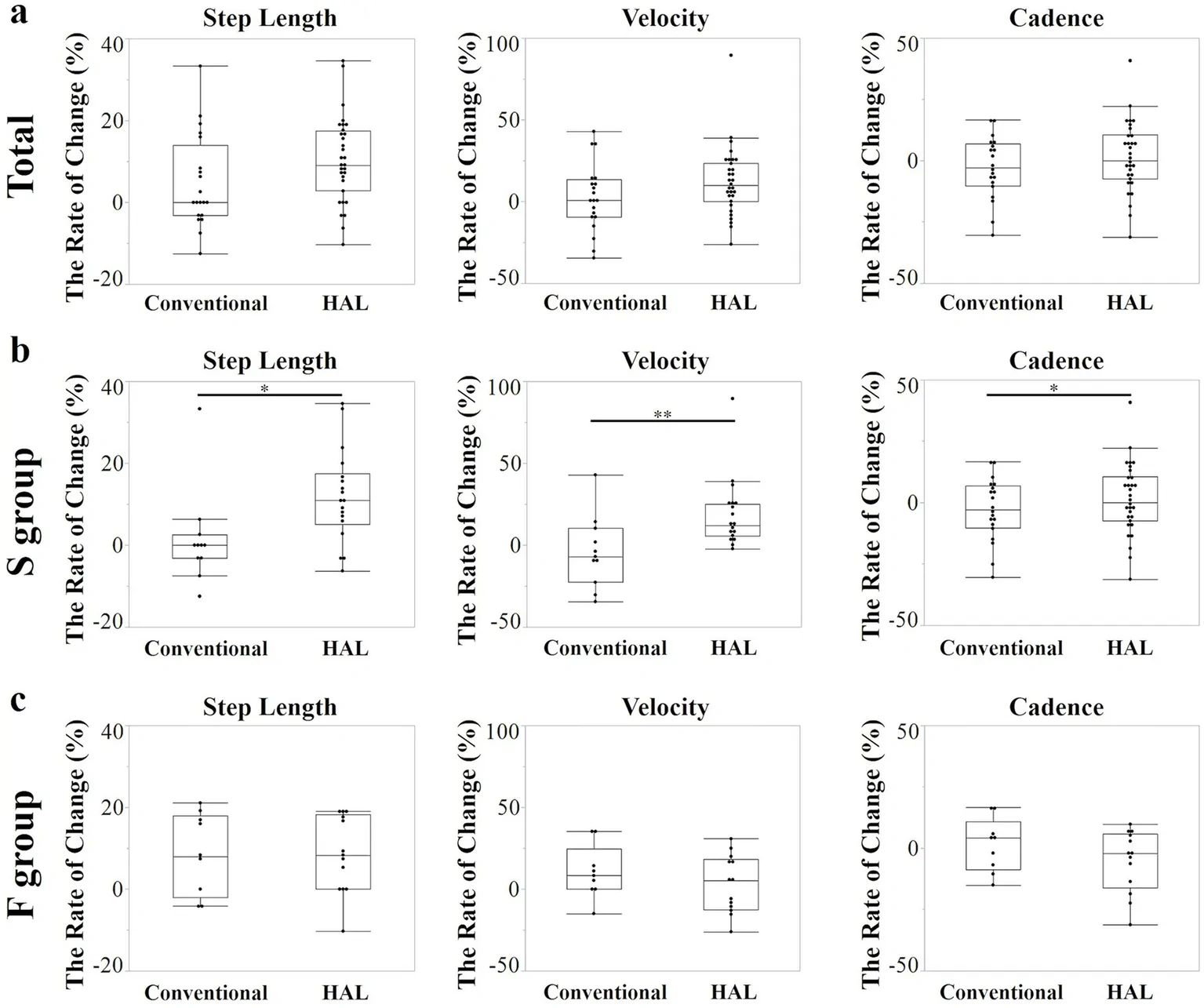
Comparisons of the rates of change between the HAL and conventional gait training groups. Comparison of the rates of change in step length, gait velocity, and cadence immediately after HAL and conventional gait training for all units **(a)**, the S group **(b)**, and the F group **(c)**. No significant differences were observed between the training methods when all units were analyzed together. Significant differences were observed in step length, gait velocity, and cadence in the S group, whereas no significant differences were found in the F group. *p < 0.05; **p < 0.01; Mann–Whitney’s U-test. S, slow; F, fast; HAL, Hybrid Assistive Limb.

## 4 Discussion

In this study, after adjustment for patient-specific differences, the corresponding pre-training value, and days from the first gait-training session, no significant differences were observed in changes in step length, velocity, or cadence between HAL and conventional gait training. In contrast, the adjusted between-condition differences in step length and velocity were numerically greater with HAL gait training; however, their 95% CIs included zero. In the within-condition analyses, step length and velocity in the 10MWT increased significantly immediately after HAL gait training, whereas no changes were observed after conventional gait training. These findings suggest that HAL gait training may have an immediate effect on gait performance in patients in the acute phase of stroke, particularly through improvements in step length and velocity; however, the superiority of HAL over conventional gait training was not demonstrated in the adjusted analysis.

Tanaka et al. (2019) reported that step length and velocity increased after HAL gait training in patients in the chronic phase of stroke. Our findings suggest that similar immediate changes may also occur after HAL gait training in the acute phase of stroke. However, given the lack of statistically significant differences between HAL and conventional gait training in the adjusted analysis, these findings should be interpreted cautiously.

Our results showed a significant negative correlation between cadence immediately before HAL gait training and its rate of change immediately after training, suggesting that the lower the cadence, the greater the immediate change in cadence after HAL gait training. Kubota et al. (2013) reported that improvement in gait velocity after 16 HAL gait training sessions was primarily due to improvement in cadence, although their study included patients with other diseases. However, in our study, no significant changes in cadence were found from immediately before to after HAL gait training. This discrepancy may be because, in some units, cadence was low before HAL gait training and further decreased immediately after training due to fatigue.

In the S group (velocity ≤ 0.4 m/s), all gait parameters showed greater improvement after HAL gait training than after conventional training. This finding from the exploratory subgroup analyses suggests that patients with lower walking velocity may be more likely to show an immediate response to HAL gait training. However, because this subgroup analysis did not account for repeated measurements within a small number of patients, a walking velocity of 0.4 m/s should not be interpreted as a definitive threshold for selecting HAL gait training. At the beginning of the series, all four patients were classified into the S group based on gait velocity. As the series progressed, gait velocities increased, and consequently, three of the four patients transitioned from the S to the F group (Figure 2b). Although the optimal initiation timing remains unclear, these observations raise the possibility that HAL gait training may be particularly beneficial early after stroke when walking ability is limited. As HAL application requires time and manpower, identifying patients who are more likely to benefit from HAL gait training may be clinically important. Further studies are warranted to clarify whether walking velocity can be used as a criterion for patient selection. In the exploratory analysis of the S group, HAL use was associated with greater improvements in step length and cadence, which may have contributed to the greater improvement in walking velocity. Early implementation of HAL gait training in patients with stroke has been shown to be effective. Degami et al. (2022) reported that the Brunnstrom stage of the lower limb and the modified Rankin Scale score improved in patients who started HAL gait training earlier than in those who started it later. Together with these previous findings, our results suggest that the potential benefits of initiating HAL gait training early after stroke warrant further investigation. However, whether repeated immediate effects accumulate and contribute to better long-term outcomes remains to be determined.

Although the physiological mechanisms underlying the immediate changes after HAL gait training remain unclear, the changes observed within a single training session may reflect transient sensorimotor facilitation and/or motor learning rather than changes in muscle strength alone. During HAL gait training, movement-related sensory information is conveyed to the central nervous system through interactive biofeedback, which may facilitate sensorimotor integration (Morishita and Inoue, 2016). Additionally, somatosensory input has been suggested to contribute to motor learning (Cauraugh and Summers, 2005). Ando et al. (2020) reported changes in interhemispheric communication following early HAL gait training, suggesting a possible neural effect of the intervention. However, because the present study did not directly assess neural activity or motor-learning processes, whether the observed immediate changes reflect transient sensorimotor facilitation, neuroplastic motor learning, or a combination of these mechanisms remains to be determined.

Early initiation of HAL gait training may offer several advantages. HAL gait training has been shown to increase walking distance during training (Nankaku et al., 2020; Wall et al., 2020). Therefore, early mobilization can be expected in patients undergoing HAL gait training. Spasticity is notably lower in the acute phase of stroke than in the chronic phase, during which it worsens and often becomes a major barrier to rehabilitation, making robot-assisted gait training more difficult to perform. This may provide a rationale for considering HAL gait training early after stroke. Combining robot-assisted training with botulinum neurotoxin injections has been reported to be effective for rehabilitation of the upper limbs (Okuno et al., 2021; Hyakutake et al., 2022). However, the larger amount of muscles in the lower limbs than in the upper limbs makes the use of botulinum neurotoxin more challenging. Moreover, as HAL gait training can modulate gait patterns (Kawamoto et al., 2015; Puentes et al., 2018a, b), it allows patients to learn proper gait patterns, and early initiation of active HAL gait training may help prevent the progression of spasticity.

This study has some limitations. First, we only examined patients in the acute phase of stroke and did not consider those in the chronic phase. Evaluation of long-term outcomes is needed to clarify the usefulness of HAL gait training in patients with stroke. Second, the number of patients was very small, and multiple measurements were obtained from each patient. Although the primary comparison accounted for patient-specific differences, the corresponding pre-training value, and days from the first gait-training session, the small number of patients limits the generalizability of the findings. Third, the subgroup analyses were exploratory and did not account for the within-patient correlation of repeated measurements; therefore, these results should be interpreted with caution. Finally, this study was non-randomized and unblinded. Further randomized prospective studies with larger sample sizes are warranted to corroborate the immediate effects of HAL gait training and identify patients who are most likely to benefit from it.

In conclusion, HAL gait training was associated with significant immediate improvements in step length and walking velocity in the within-condition analysis; however, the adjusted between-condition analysis did not demonstrate a statistically significant difference between HAL and conventional gait training. Exploratory analyses suggested that patients with lower walking velocity may show a greater immediate response to HAL gait training. These preliminary findings support further investigation of early HAL gait training in patients in the acute phase of stroke, particularly those with limited walking ability.

## Data Availability

The original contributions presented in the study are included in the article/Supplementary material, further inquiries can be directed to the corresponding author.
