# Supplementary material for: Immediate effect of gait training using the hybrid assistive limb in patients in the acute phase of stroke: a prospective pilot study
Source: Front Neurorobot. 2026 Oct 23;20:1985500. doi: 10.3389/fnbot.2026.1985500 (PMC13739807; doi:10.3389/fnbot.2026.1985500)
Supplement: Supplementary file 1 [file Data_Sheet_1.docx]

Supplementary Material

# 1 Supplementary Figures and Tables

# 1.1 Supplementary Tables

**Supplementary Table 1: Individual session-level data for all patients**

| Patient | Gait Training | Gait Training Date | Step length | | | Velocity | | | Cadence | | |
| --- | --- | --- | --- | --- | --- | --- | --- | --- | --- | --- | --- |
|  |  |  | pre | post | Δ | pre | post | Δ | pre | post | Δ |
| 1 | H | 1 | - | - | - | - | - | - | - | - | - |
| 1 | C | 2 | - | - | - | - | - | - | - | - | - |
| 1 | H | 3 | 19.2 | 23.8 | 4.6 | 0.13 | 0.18 | 0.05 | 40.0 | 44.2 | 4.2 |
| 1 | C | 4 | 20.8 | 20.8 | 0.0 | 0.19 | 0.19 | 0.00 | 53.3 | 54.3 | 1.0 |
| 1 | H | 5 | 22.2 | 20.8 | -1.4 | 0.17 | 0.18 | 0.01 | 45.0 | 51.4 | 6.4 |
| 1 | H | 8 | 23.8 | 25.6 | 1.8 | 0.23 | 0.22 | -0.01 | 57.3 | 52.0 | -5.3 |
| 1 | C | 9 | 29.4 | 29.4 | 0.0 | 0.31 | 0.22 | -0.10 | 63.8 | 44.3 | -19.4 |
| 1 | H | 10 | 23.8 | 27.8 | 4.0 | 0.23 | 0.25 | 0.02 | 57.3 | 54.0 | -3.3 |
| 1 | C | 11 | 23.8 | 20.8 | -3.0 | 0.26 | 0.17 | -0.09 | 66.3 | 49.7 | -16.7 |
| 1 | H | 12 | 27.0 | 31.3 | 4.2 | 0.26 | 0.26 | 0.00 | 58.4 | 50.5 | -7.9 |
| 1 | H | 15 | 28.6 | 32.3 | 3.7 | 0.28 | 0.29 | 0.01 | 58.3 | 53.1 | -5.2 |
| 1 | C | 16 | 25.6 | 26.3 | 0.7 | 0.25 | 0.23 | -0.02 | 58.5 | 53.0 | -5.5 |
| 1 | H | 17 | 34.5 | 37.0 | 2.6 | 0.59 | 0.43 | -0.15 | 102.4 | 70.4 | -31.9 |
| 1 | C | 18 | 32.3 | 38.5 | 6.2 | 0.50 | 0.56 | 0.06 | 93.0 | 86.7 | -6.3 |
| 1 | H | 19 | 38.5 | 34.5 | -4.0 | 0.53 | 0.45 | -0.07 | 82.1 | 79.1 | -3.0 |
| 2 | H | 1 | - | - | - | - | - | - | - | - | - |
| 2 | C | 2 | - | - | - | - | - | - | - | - | - |
| 2 | H | 3 | - | - | - | - | - | - | - | - | - |
| 2 | C | 4 | 27.0 | 25.0 | -2.0 | 0.29 | 0.23 | -0.07 | 65.3 | 54.5 | -10.7 |
| 2 | H | 5 | 23.3 | 26.3 | 3.1 | 0.21 | 0.23 | 0.02 | 53.8 | 53.0 | -0.7 |
| 2 | H | 8 | 27.8 | 29.4 | 1.6 | 0.28 | 0.29 | 0.02 | 60.0 | 60.0 | 0.0 |
| 2 | C | 9 | 29.4 | 29.4 | 0.0 | 0.31 | 0.34 | 0.03 | 63.8 | 70.3 | 6.6 |
| 2 | H | 10 | 33.3 | 35.7 | 2.4 | 0.33 | 0.42 | 0.08 | 60.0 | 70.0 | 10.0 |
| 2 | C | 11 | 32.3 | 32.3 | 0.0 | 0.40 | 0.38 | -0.02 | 74.4 | 71.5 | -2.9 |
| 2 | H | 12 | 33.3 | 32.3 | -1.1 | 0.40 | 0.42 | 0.02 | 72.0 | 77.5 | 5.5 |
| 2 | H | 15 | 33.3 | 32.3 | -1.1 | 0.36 | 0.40 | 0.04 | 64.3 | 74.4 | 10.1 |
| 2 | C | 16 | 32.3 | 31.3 | -1.0 | 0.36 | 0.32 | -0.03 | 66.4 | 61.9 | -4.5 |
| 2 | H | 17 | 32.3 | 35.7 | 3.5 | 0.40 | 0.48 | 0.08 | 74.4 | 80.0 | 5.6 |
| 2 | C | 18 | 33.3 | 32.3 | -1.1 | 0.33 | 0.30 | -0.03 | 60.0 | 56.4 | -3.6 |
| 2 | H | 19 | 33.3 | 37.0 | 3.7 | 0.40 | 0.43 | 0.03 | 72.0 | 70.4 | -1.6 |
| 3 | H | 1 | - | - | - | - | - | - | - | - | - |
| 3 | C | 2 | - | - | - | - | - | - | - | - | - |
| 3 | H | 3 | 20.8 | 27.8 | 6.9 | 0.24 | 0.29 | 0.06 | 68.6 | 63.5 | -5.0 |
| 3 | C | 4 | 31.3 | 41.7 | 10.4 | 0.33 | 0.48 | 0.14 | 64.0 | 68.6 | 4.6 |
| 3 | H | 5 | 33.3 | 40.0 | 6.7 | 0.40 | 0.56 | 0.16 | 72.0 | 83.3 | 11.3 |
| 3 | H | 8 | 38.5 | 41.7 | 3.2 | 0.67 | 0.59 | -0.08 | 104.0 | 84.7 | -19.3 |
| 3 | C | 9 | 34.5 | 37.0 | 2.6 | 0.50 | 0.53 | 0.03 | 87.0 | 85.3 | -1.7 |
| 3 | H | 10 | 40.0 | 47.6 | 7.6 | 0.71 | 0.83 | 0.12 | 107.1 | 105.0 | -2.1 |
| 3 | C | 11 | 43.5 | 41.7 | -1.8 | 0.71 | 0.71 | 0.00 | 98.6 | 102.9 | 4.3 |
| 3 | H | 12 | 41.7 | 45.5 | 3.8 | 0.91 | 0.77 | -0.14 | 130.9 | 101.5 | -29.4 |
| 3 | H | 15 | 40.0 | 47.6 | 7.6 | 0.71 | 0.83 | 0.12 | 107.1 | 105.0 | -2.1 |
| 3 | C | 16 | 37.0 | 40.0 | 3.0 | 0.63 | 0.71 | 0.09 | 101.3 | 107.1 | 5.9 |
| 3 | H | 17 | 50.0 | 52.6 | 2.6 | 1.00 | 0.91 | -0.09 | 120.0 | 103.6 | -16.4 |
| 3 | C | 18 | 43.5 | 52.6 | 9.2 | 0.77 | 0.83 | 0.06 | 106.2 | 95.0 | -11.2 |
| 3 | H | 19 | 47.6 | 55.6 | 7.9 | 0.83 | 1.00 | 0.17 | 105.0 | 108.0 | 3.0 |
| 4 | H | 1 | - | - | - | - | - | - | - | - | - |
| 4 | C | 2 | - | - | - | - | - | - | - | - | - |
| 4 | H | 3 | 27.0 | 27.8 | 0.8 | 0.23 | 0.29 | 0.06 | 50.5 | 61.7 | 11.3 |
| 4 | C | 4 | 29.4 | 31.3 | 1.8 | 0.31 | 0.36 | 0.04 | 63.8 | 68.6 | 4.8 |
| 4 | H | 5 | 27.8 | 30.3 | 2.5 | 0.27 | 0.33 | 0.06 | 58.4 | 66.0 | 7.6 |
| 4 | H | 8 | 28.6 | 38.5 | 9.9 | 0.28 | 0.53 | 0.25 | 58.3 | 82.1 | 23.8 |
| 4 | C | 9 | 34.5 | 40.0 | 5.5 | 0.43 | 0.59 | 0.15 | 75.7 | 88.2 | 12.6 |
| 4 | H | 10 | 35.7 | 35.7 | 0.0 | 0.50 | 0.53 | 0.03 | 84.0 | 88.4 | 4.4 |
| 4 | C | 11 | 40.0 | 40.0 | 0.0 | 0.59 | 0.50 | -0.09 | 88.2 | 75.0 | -13.2 |
| 4 | H | 12 | 37.0 | 43.5 | 6.4 | 0.50 | 0.63 | 0.13 | 81.0 | 86.3 | 5.3 |
| 4 | H | 15 | 45.5 | 45.5 | 0.0 | 0.67 | 0.63 | -0.04 | 88.0 | 82.5 | -5.5 |
| 4 | C | 16 | 43.5 | 41.7 | -1.8 | 0.63 | 0.63 | 0.00 | 86.3 | 90.0 | 3.8 |
| 4 | H | 17 | 40.0 | 47.6 | 7.6 | 0.59 | 0.77 | 0.18 | 88.2 | 96.9 | 8.7 |
| 4 | C | 18 | 35.7 | 41.7 | 6.0 | 0.43 | 0.59 | 0.15 | 73.0 | 84.7 | 11.7 |
| 4 | H | 19 | 41.7 | 41.7 | 0.0 | 0.59 | 0.63 | 0.04 | 84.7 | 90.0 | 5.3 |

Δ indicates change. C, conventional gait training; H, Hybrid Assistive Limb (HAL) gait training.

**Supplementary Table 2.** Step length, velocity, and cadence immediately before conventional and HAL gait training in the total, S, and F groups

|  |  | Total | | | S group | | | F group | | |
| --- | --- | --- | --- | --- | --- | --- | --- | --- | --- | --- |
|  |  | HAL  (n = 31) | Conventional  (n = 20) | p | HAL  (n = 18) | Conventional  (n = 11) | p | HAL  (n = 13) | Conventional  (n = 9) | p |
| Records of before rehabilitation | Step length (cm) | 33.3 (19.2–50.0) | 32.3 (20.8–43.5) | 0.9461 | 27.8 (19.2–33.3) | 29.4 (20.8–33.3) | 0.7005 | 40.0 (34.5–50.0) | 37.0 (32.3–43.5) | 0.2675 |
|  | Velocity (m/s) | 0.40 (0.13–1.00) | 0.38 (0.19–0.77) | 0.8772 | 0.28 (0.13–0.40) | 0.31 (0.19–0.40) | 0.3785 | 0.67 (0.50–1.00) | 0.59 (0.43–0.77) | 0.1677 |
|  | Cadence (steps/min) | 72.0 (40.0–130.9) | 69.7 (53.3–106.2) | 0.7066 | 58.4 (40.0–74.4) | 63.8 (53.3–74.4) | 0.1915 | 102.4 (81.0–130.9) | 88.2 (73.0–106.2) | 0.2423 |

S, slow; F, fast; HAL, Hybrid Assistive Limb

**Supplementary Table 3.** Distribution of S and F groups for each patient

|  | HAL | | | Conventional | | |
| --- | --- | --- | --- | --- | --- | --- |
|  | Total | S group | F group | Total | S group | F group |
| Case 1 | 8 | 6 | 2 | 5 | 4 | 1 |
| Case 2 | 7 | 7 | 0 | 5 | 5 | 0 |
| Case 3 | 8 | 2 | 6 | 5 | 1 | 4 |
| Case 4 | 8 | 3 | 5 | 5 | 1 | 4 |
| Total | 31 | 18 | 13 | 20 | 11 | 9 |

S, slow; F, fast; HAL, Hybrid Assistive Limb
